# Education associated with a delayed onset of terminal decline

**DOI:** 10.1093/ageing/aft150

**Published:** 2013-10-17

**Authors:** Graciela Muniz Terrera, Thais Minett, Carol Brayne, Fiona E. Matthews

**Affiliations:** 1 MRC Lifelong Health and Ageing Unit, UCL, 33 Bedford Place, London WC1B 5JU, UK; 2 Department of Public Health and Primary Care, Institute of Public Health, University of Cambridge, Cambridge CB2 2SR, UK

**Keywords:** terminal decline, MMSE, change points, older people

## Abstract

**Background**: the terminal decline hypothesis suggests an acceleration in the rate of loss of cognitive function before death. Evidence about the association of educational attainment and the onset of terminal decline is scarce.

**Objective**: to investigate the association of education with the onset of terminal decline in global cognitive function measured by Mini Mental State Exam (MMSE) scores.

**Subjects**: deceased participants of the Cambridge City over 75 Cohort Study who were interviewed at about 2, 7, 9, 13, 17 and 21 years after baseline.

**Methods**: regular and Tobit random change point growth models were fitted to MMSE scores to identify the onset of terminal decline and assess the effect of education on this onset.

**Results**: people who left school at an older age had a delayed onset of terminal decline. Thus better educated individuals experience a slightly shorter period of faster decline before death.

**Conclusion**: an important finding emerging from our work is that education does appear to delay the onset of terminal decline, although only by a limited amount.

## Introduction

The world population is ageing rapidly. The identification of mechanisms that may postpone the onset of disability is important as this postponement may lead to a ‘compression of morbidity’ so that individuals experience a shorter span between death and the onset of chronic disease, disability and loss of independence [1].

The terminal decline hypothesis suggests an accelerated decline in cognitive function before death [2]. Evidence is inconsistent, varying by ability examined. Some did not find an accelerating change of primary and episodic memory, word recognition, verbal and visuospatial ability, MMSE scores and perceptual speed [3–5]. Others reported constant rate of decline for Block Design score, an ‘age-vulnerable’ activity, and accelerating rate of decline in Information scores, an ‘age-maintained’ ability [6]. Results about the onset of terminal decline also varied by study and ability, ranging from 2.7 years before death for perceptual speed [7] to 14.8 years for spatial ability [8].

The brain reserve hypothesis postulates ‘the existence of individual differences in how people process tasks allows some to cope better than others with brain pathology’ [9], suggesting that individuals most exposed to more demanding cognitive activities over their lifespan can compensate for more brain damage before deterioration becomes clinically manifest than individuals less exposed to such intellectual activities.

Education is often considered as a proxy for brain reserve. Evidence of its effect on the terminal decline process is divergent. Laukka *et al*. [4] reported that, in the proximity of death, more educated individuals experienced a slower decline in Block Design scores than less educated individuals; whilst others who examined change in verbal abilities, MMSE, information and block design scores reported an association of education with performance but not with rate of decline [4, 6, 10].

In a report of the association of education with the onset of terminal decline, the onset of terminal decline was found to vary by level of education for some but not all measures [11].

In the Cambridge City over 75 Cohort Study (CC75C), we examined the terminal decline hypothesis previously [12, 13], but models used did not allow us to test for associations of risk factors with this onset [14].

Here, we investigate whether education, a key proxy for life course brain ageing related to health has any association with the timing of the onset of terminal decline.

## Method

### Sample

The CC75C study (http://www.cc75c.group.cam.ac.uk) is a population-based study which began in 1985. [15]. People aged at least 75 years old or older in 1985 registered at any of five selected primary care practices, and one in three from a sixth practice in the Cambridge City area were recruited. Cognitive status was assessed using the MMSE, a brief test of global function that takes integer values in the 0–30 interval, with high scores indicating good cognition [16]. When a question was not asked or was not applicable due to sensory or physical impairment, the item was scored as zero (impaired) and included in the calculation of the final MMSE score.

After baseline, interviews were conducted on all the survivors at about 2, 7, and 9, 13, 17 and 21 years later.

Date of death was obtained from the National Health Service Central Register. Most study participants have died (only five alive at time of analysis). On average, the interval between the last interview and death was 2.8 years (SD = 2.6, range = [0.7, 19.7]). The mean age at study entry was 81 years old and at death was 88 years old. Women accounted for 65% of the deceased sample (see Table 1 for descriptive characteristics of CC75C study participants).

**Table 1.** Descriptive characteristics of Cambridge City over 75 Cohort study participants

Interview wave							
Mean (SD) (range)	First (*n* = 2078)	Second (*n* = 1141)	Third (*n* = 640)	Fourth (*n* = 379)	Fifth (*n* = 166)	Sixth (*n* = 76)	Seventh (*n* = 15)
MMSE	25.4 (4.5) [0, 30]	24.3 (4.3) [0, 30]	22.7 (5.8) [0, 30]	22.0 (6.6) [0, 30]	21.4 (6.0) [5, 30]	18.9 (7.2) [0, 30]	16.3 (7.4) [0, 27]
Years to death	7.2 (5.0) [0.04, 22.2]	6.6 (4.5) [0.01,19.7]	5.3 (3.7) [0.01, 15.7]	4.5 (3.0) [0.1, 12.7]	3.5 (2.3) [0.1, 9.3]	2.2 (1.5) [0.05, 5.6]	0.8 (0.5) [0.1, 2]
Time between death and last interview (years)	3.5 (3.3) [0.01, 19.7]	2.7 (2.5) [0.01,19.5]	2.3 (1.9) [0.01, 15.6]	2.4 (1.8) [0.01, 10.3]	2.1 (1.4) [0.05, 8.4]	1.5 (1.2) [0.01, 4.6]	0.8 (0.5) [0.01, 1.9]
Women (%)	1355 (65%)	746 (65%)	437 (68%)	267 (70%)	120 (72%)	60 (78%)	13 (86%)
Physical impairment at study entry^a^ (%)	1051 (50%)	489 (43%)	238 (37%)	123 (32%)	44 (26%)	18 (24%)	3 (20%)
Age at which individual left school	14.8 (2.1) [10, 26]	14.9 (2.2) [10, 26]	15.1 (2.3) [10, 26]	15.1 (2.4) [10, 26]	15.1 (2.0) [10, 26]	15.2 (2.0) (Fries 245–50)	15.3 (2.4) [13, 21]
Age at death	88.5 (5.4) [75.2, 106.6]	89.7 (5.0) [78.1,106.6]	91.6 (4.4) [81.8, 106.6]	92.7 (5.9) [85.0, 106.6]	94.6 (3.2) [88.7, 106.6]	94.6 (2.0) [91.6, 102.1]	98.2 (1.5) [95.7, 100.7]

^a^Impairment defined as present when the individual could not walk unaided around town or the block.

Education was measured according to the age individuals left school. On average, individuals left school aged 14.8 years (SD = 2.2, range = [0, 28]).

At baseline, functional status was measured using the self-reported Instrumental Activities of Daily Living (IADL) or Activities of Daily Living (ADL). We derived three indicator variables to account for physical impairment at baseline (1 = if person unable to walk unaided around the block, 0 = otherwise), cognitive impairment at baseline (1 = if MMSE ≤ 23, 0 = otherwise) and gender (1 = women, 0 = men). Age at death and education were centred using their mean values.

Supplementary data are available in *Age and Ageing* online Figure 1A1 in Appendix 1 show MMSE trajectories (pale blue) plotted as a function of distance to death for the group of individuals who left school aged older than the mean age at which CC75C study participants left school (14.8 years) (left panel), and for individuals who left school aged younger than 14.8 years (right panel) and model estimated mean curves for each group (thick black lines).

### Statistical methods

We fitted regular and Tobit random change point models (to account for possible ceiling/floor effects) to describe MMSE changes as a function of years to death (in negative values counting time in years before death) [17].

We considered models with two linear phases, before and after the onset of terminal decline and set the origin at 2 years before death. The onset of faster change (change point) was modelled as a random effect and its association with education examined.

Rate of change before and after the change point was modelled as functions of population mean parameters, baseline physical and cognitive impairment, age at death, gender and education. Hence, the two linear slopes represent rate of decline before and after the change point for a man physically and cognitively able at study entry, who had left school aged 14.8 years old and died aged 88 years old. (see Supplementary data are available in *Age and Ageing* online Appendix 2 for a mathematical formulation of the models).

Models were fitted in a Bayesian framework using WinBUGS, a package that uses Markov chain MonteCarlo methods to estimate model parameters [18]. Deviance Information Criterion (DIC) values were obtained to compare model fit [19]. Model fit was assessed visually (see Supplementary data are available in *Age and Ageing* online Appendix 3 for some of the graphs inspected to assess model fit). Low values indicate better fit. Missing data were assumed to be missing at random.

## Results

Most individuals left school aged younger than 15 years old (69%). At baseline, 43% of the individuals were physically impaired, almost one in five had cognitive impairment (19%) and 7% of the sample scored 30 on the MMSE. Only 2% were still at ceiling on the second interview.

Although DIC values obtained from the Tobit (DIC = 51518.5) and regular models (DIC = 51524.3) were similar, they suggested that the Tobit model supported the data best. Hence, we report results for the Tobit model.

The onset of terminal decline was estimated, on average, at 6.2 years before death (SD = 0.2). Results of the model fitted are shown in Table 2.

**Table 2.** Posterior mean estimates, standard errors and 95% credible interval of cognitive performance 2 years before death, the onset of terminal decline, rate of decline before and after this onset, change in rate of decline after the onset of terminal decline and of the effect of risk factors on these parameters

Fixed effects	Estimate (SD)	95% credible interval
Cognitive status 2 years before death	23.1 (0.2)	[22.7, 23.5]
Rate of decline before onset of terminal decline	−0.12 (0.02)	[−0.16, −0.08]
Age at death	0.01 (0.01)	[0.007, 0.01]
Physical impairment at study entry	−0.02 (0.02)	[−0.05, 0.01]
Women	−0.02 (0.01)	[−0.04, −0.004]
Education	0.2 (0.03)	[0.1, 0.3]
Cognitively impaired at baseline	−5.2 (0.2)	[−5.7, −4.8]
Rate of decline after onset of terminal decline	−0.91 (0.05)	[−1.0, −0.8]
Age at death	−0.06 (0.006)	[−0.007, − 0.05]
Physical impairment at study entry	−0.04 (0.05)	[−0.15, 0.07]
Women	0.10 (0.03)	[0.04, 0.16]
Education	−0.005 (0.001)	[−0.07, −0.003]
Cognitively impaired at baseline	−0.5 (0.08)	[−0.68, −0.36]
Onset of terminal decline	−6.2 (0.2)	[−6.6, −5.7]
Education	0.4 (0.08)	[0.2, 0.5]
Variance random effects		
Rate of change before onset of terminal decline	0.02 (0.003)	[0.02, 0.03]
Rate of change after onset of terminal decline	0.5 (0.05)	[0.4, 0.6]

MMSE scores of a physically and non-cognitively impaired man who left school aged 15 years old and died aged 88 years, declined at a rate of 0.12 (SD = 0.021) points per year closer to death before the change point. This rate increased to 0.91 (SD = 0.06) points per year closer to death after the change point. Two years before death, cognitive performance was estimated at 23.1 (SD = 0.2).

Older age at death and baseline cognitive impairment were associated with rate of decline before and after the change point. Women declined faster than men before the change point and at a slower rate after it. Physical impairment was not associated with rate of change, either before or after the onset of faster decline.

The correlation between the slope before and after the change point decline was −0.54, a value that suggests that individuals whose rate of change before the onset of terminal decline is positive decline faster once the terminal decline phase started.

**Figure 1.**
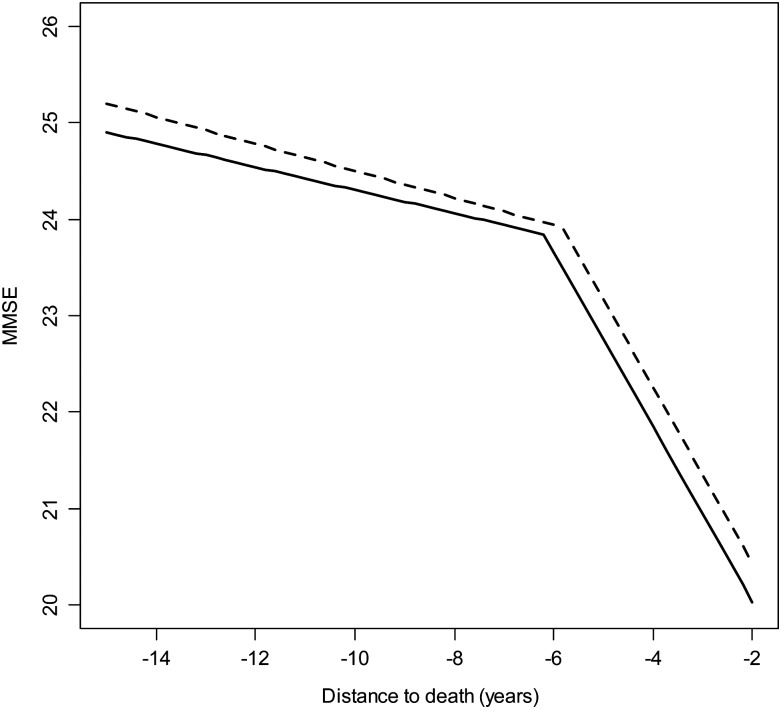
Estimated trajectories of MMSE scores prior to death for a physically able man at study entry, who died aged 88 years old and had left school aged 15 years old (solid line) and for a man of similar characteristics who left school aged 16 (thick dashed line).

Individuals who left school at an older age had a later onset of faster decline, although the onset was delayed by only 0.4 years for more educated individuals. Figure 1 shows the model estimated MMSE trajectory for a man with reference values in all model covariates and for a man of similar characteristics who left school aged 16 years old.

Education was associated with rate of decline before the change point, but not after it (see Table 2). Individuals who stayed an extra year at school declined at a slightly faster rate than those who left school at a younger age. Nonlinear effects of education on the onset of terminal decline were investigated by adjusting the onset of faster decline by education and education squared, but nonlinear effects of education were not found in the data.

## Discussion

Our results indicate that the onset of terminal decline in global cognitive function measured using the MMSE occurred at 6.2 years before death. Education was associated with a small delay of the onset of terminal decline although by a small amount. This result suggests that it may be possible to delay the onset of terminal decline (some of which will be manifest as dementia) through extending and improving an individual's education. However, this terminal decline process might not be avoided but only shortened. Our findings agree with the brain reserve hypothesis supporting compensation for a fundamental underlying process which is associated with mortality. This fits well with findings from similar studies [20].

### Strengths and limitations

Our investigation benefited from the fact that the cohort examined was followed up to near extinction, from the application of a methodology that allowed us to relax the assumption of a common onset of terminal decline and to examine the association of education with the location of the onset of accelerated decline.

Study limitations include the inability to consider cause of death and measures of health status which may influence patterns of terminal decline; the use of a limited and simple measure of educational attainment due to lack of other information about education and engagement in cognitive challenging activities; and the separation of interviews may have limited our ability to better capture change close to death, as it is possible that we did not have enough observations close to death for those individuals who died between interviews. Also, some individuals dropped out the study early and died at a later date. However, in an examination of the impact of the length of time elapsed between death and dropout on model results, we conducted a sensitivity analysis excluding from analysis individuals who were last interviewed 5 (79% of sample), 7 (88% of sample) and 10 years before death (93% of sample). Results about the effect of education on the onset of terminal decline were found to be consistent.

Further, we were only able to examine change in MMSE scores, despite it might not be the most sensitive measure to test terminal decline and were unable to analyse domain specific MMSE subscores as they were unavailable to us.

The MMSE tests a diversity of skills that are likely to be affected in different ways by the terminal decline process and on which education might have a different impact. MMSE ceiling and floor effects were accounted for by using a Tobit model, that marginally improved model fit compared with the regular growth model and produced very similar estimates of fixed and random effects. This may be explained by the low percentage of individuals at ceiling in our study (7%). Another eventual limitation is that questions not asked due to sensory or physical impairment were scored as zero in the final MMSE score, which may explain the positive association of cognitive impairment and rate of decline before the onset of terminal decline.

Education was associated with a delayed onset of terminal decline in the population. Hence, the time spent in the terminal phase by more educated individuals is shorter than for less educated individuals, a finding that agrees with the health expectancy literature where a reduction in the number of years spent with impairment with increasing education was reported [21]. Our findings support the compression of morbidity idea, as discussed by Fries [22] who proposed that morbidity might be compressed into a shorter span between the increasing age at onset of disability and the fixed occurrence of death. Although detectable, the magnitude of the compression is limited at the individual level but relevant at population as public policies to improve education in the population could reduce the time spent in the critically declining and more resource consuming final stages of life.

Evidence about the effect of education in the final years of the oldest old is scarce. Education was reported to affect the onset of terminal decline in processing speed, episodic memory and global cognition in different ways [11]. In this study, individuals in the lower education group had a delayed onset of terminal decline, but they declined at a faster rate in the terminal decline phase. For the other two measures, education did not significantly modify the onset of terminal decline. The inconsistency of results across measures was explained by the different cerebral and biological deterioration processes that could occurring before death. Others have reported a positive association between education and performance in some cognitive abilities but not with rate of decline before death [4, 6, 13]. Differences in features of the samples, ability, tests and methodologies employed may explain these divergent results. For instance, Wilson *et al*. [23] examined a composite measure of cognition from a sample of volunteers, Laukka *et al*. [4] examined MMSE scores from population and community-based samples and Piccinin *et al*. and Johansson *et al*. [6, 10] investigated a range of cognitive abilities from a Swedish study of twins. Although most studies used random effects models [24], there are important methodological differences in the models considered that may contribute to the diversity of findings. For instance, whilst some used quadratic latent growth models to describe accelerating cognitive change [6, 10], others [5, 23] used change point models that estimate a common onset for all individuals in the sample, an assumption unlikely to be fulfilled in heterogeneous sample. Furthermore, some studies [13, 23] used a serial combination of time-to-event time metrics instead of a single time-to-death metric to model change, resulting in the alignment of individuals according to processes different from death. This may affect results as it has been demonstrated that time-to-death is a better indicator of change in the proximity of death than a combined time metric [25].

It has been argued that years of formal education or age at which individuals left school may not be a good proxy for brain reserve [26], and that this information should be complemented with information about quality of education and other indicators of cultural experiences. However, in the CC75C, a UK population-based study of ageing, where individuals have relatively homogeneous early life educational experiences and ethnic backgrounds, this measure appears to have a clear evident association with factors which have an influence even in the years before death.

The key finding emerging from this cohort in which almost all individuals have died is that education delayed the onset of terminal decline. This finding has implications for policy development early in life and for planning for the end of life.

## Supplementary data

Supplementary data mentioned in the text are available to subscribers in *Age and Ageing* online.

**Key points**

- Education appears to slightly delay the onset of terminal decline.
- Thus better educated individuals experience a slightly shorter period of faster decline before death.
- Our results are in support of the compression of morbidity hypothesis.

## Funding

This work was supported by UK Medical Research Council CDA WBS U.1052.00.013.00003 (Graciela Muniz Terrera) and UK Medical Research Council WBS U.1052.00.013.00001 (Fiona Matthews). CC75C is part of the Cambridgeshire and Peterborough CLARHC.

## Supplementary Material

Supplementary Data
