# Supplementary material for: Education associated with a delayed onset of terminal decline
Source: Age Ageing. 2013 Oct 17;43(1):26–31. doi: 10.1093/ageing/aft150 (PMC3861340; doi:10.1093/ageing/aft150)
Supplement: Supplementary Data [file supp_43_1_26__index.html]

Education associated with a delayed onset of terminal decline — Supplementary Data 

# Education associated with a delayed onset of terminal decline

## Supplementary Data

Supplementary Data

**Files in this Data Supplement:**

- Supplementary Data - Doc file
