# Supplementary material for: Education associated with a delayed onset of terminal decline
Source: Age Ageing. 2013 Oct 17;43(1):26–31. doi: 10.1093/ageing/aft150 (PMC3861340; doi:10.1093/ageing/aft150)
Supplement: Supplementary Data [file supp_aft150_aft150supp.doc]

Appendix 1.

Figure 1A1. MMSE observed and predicted trajectories plotted as a function of distance to death for the group of individuals who left school at an age younger than 14.8 years, the average age at which CC75C study participants left school and for individuals who left school at an older age.


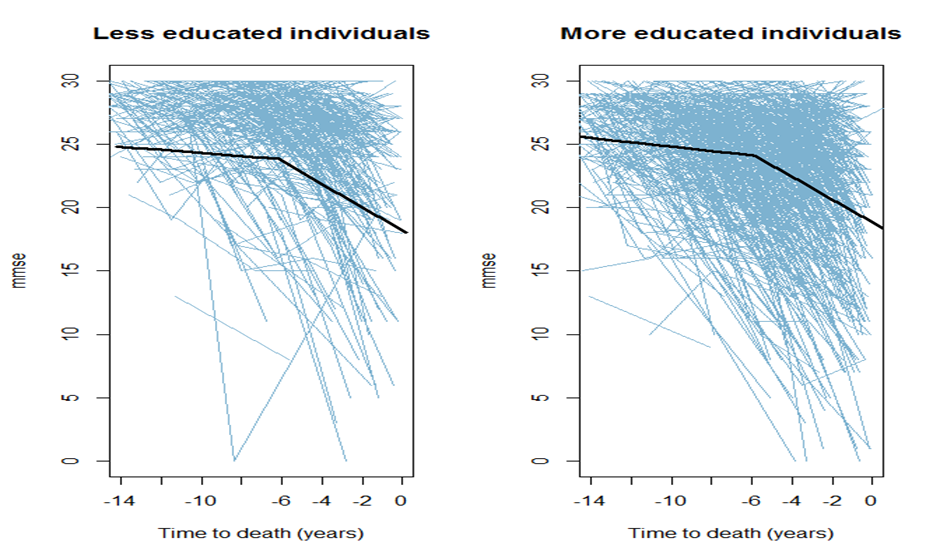


Appendix 2

The mathematical formulation of the regular model fitted is:

$$\begin{matrix} Y_{it}^{*}=(\alpha_{0i}+\alpha_{1i}T_{it})*I_{(T_{it}<\tau_{i})}+((\alpha_{0i}+\alpha_{1i}\tau_{i})+\alpha_{2i}T_{it})*I_{(T_{it}>=\tau_{i})}+\varepsilon_{it} \\ \alpha_{0i}=\alpha_{00}+\upsilon_{0i} \\ \alpha_{1i}=\alpha_{11}+\beta_{1}^{1}\text{Sex}_{i}+\beta_{1}^{2}\text{Phys Imp.}_{i}+\beta_{1}^{3}\text{Cog Imp}_{i}+\beta_{1}^{4}\text{Educ}_{i}+\beta_{1}^{5}\text{Age Death}_{i}+\upsilon_{1i} \\ \alpha_{2i}=\alpha_{22}+\beta_{2}^{1}\text{Sex}_{i}+\beta_{2}^{2}\text{Phys Imp.}_{i}+\beta_{2}^{3}\text{Cog Imp}_{i}+\beta_{2}^{4}\text{Educ}_{i}+\beta_{2}^{5}\text{Age Death}_{i}+\upsilon_{2i} \\ \tau_{i}=\tau+\beta_{3}\text{Educ}_{i}+\upsilon_{3i} \end{matrix}$$

where $Y_{it}^{^{*}}$ represents the latent outcome variable for individual *i* at time *t;* $T_{it}$ is the number of years before death from interview *t* for individual *i;* and $I$is an indicator function that takes the value of 1 if its argument is positive and zero otherwise. The latent variable $Y^{*}$is such that $Y^{*}=Y$ if $Y^{*}<30$, with $Y$ the obser ved MMSE score; and $Y=30$ if $Y^{*}>=30$. Parameters$\alpha_{1i}$ and $\alpha_{2i}$represent rate of change before and after the onset of more rapid decline for individual *i*, both modelled as functions of mean parameters $\alpha_{11}$and $\alpha_{22}$ respectively. $\beta_{1}^{k}$ and $\beta_{2}^{k}$ for k=1,..,5 represent the effect of the risk factors on rate of change before and after the onset of faster decline respectively. The onset of faster decline for each individual is represented as $\tau_{i}$, which is modelled as a funcion of a mean change point adjusted for education.

Residuals $\upsilon_{1i}$ ,$\upsilon_{2i}$ and $\upsilon_{3i}$are assumed to be independent normally distributed and independent of the error$\varepsilon_{it}$ for all values of *t* and *i.*

In a Tobit growth model, true scores $Y_{it}^{*}$ are modelled as in the previous equations, with true scores defined as $Y_{it}^{*}=Y_{it}$for $Y_{it}^{*}<c,$ and $Y_{it}=c$ for $Y_{it}^{*}>=c$ where c is the ceiling effect.

Appendix 3. Plots used for visual assessment of model fit.

Fig 1A3: Scatter plot of standardized residuals versus fitted values (left panel) and Normal Probability plot of the standardized residuals (right plot)

Fig2 and Fig 3A3: Box plots of level 2 residuals corresponding to rate of decline before and after the change point of a random sample of 50 CC75C study participants

The model was fitted using Bayesian estimation; hence, the standardized residuals presented in the graphs used to examine model fit are really single realization based on a single draw of the model parameters. As shown in the box plots presented in Fig 4A, standardized residuals are random quantities with distributions. To produce the graphs depicted in Fig 1A, we considered the mean of the standardized residuals.

1. Fries JF. Aging, natural death, and the compression of morbidity. The New England journal of medicine. 1980 Jul 17;303(3):130-5.

2. Riegel KF, Riegel RM. Development, drop, and death. Developmental Psychology; Developmental Psychology. 1972;6(2):306.

3. Ghisletta P. Application of a Joint Multivariate Longitudinal–Survival Analysis to Examine the Terminal Decline Hypothesis in the Swiss Interdisciplinary Longitudinal Study on the Oldest Old. The Journals of Gerontology Series B: Psychological Sciences and Social Sciences. 2008;63(3):P185-P92.

4. Laukka EJ, MacDonald SWS, Bäckman L. Contrasting cognitive trajectories of impending death and preclinical dementia in the very old. Neurology. 2006;66(6):833-8.

5. Laukka EJ, MacDonald SWS, Bäckman L. Terminal-decline effects for select cognitive tasks after controlling for preclinical dementia. American Journal of Geriatric Psych. 2008;16(5):355-65.

6. Piccinin AM, Muniz G, Matthews FE, Johansson B. Terminal decline from within-and between-person perspectives, accounting for incident dementia. The Journals of Gerontology Series B: Psychological Sciences and Social Sciences. 2011;66(4):391.

7. Wilson RS, Beckett LA, Bienias JL, Evans DA, Bennett DA. Terminal decline in cognitive function. Neurology. 2003;60(11):1782-7.

8. Thorvaldsson V, Hofer SM, Berg S, Skoog I, Sacuiu S, Johansson B. Onset of terminal decline in cognitive abilities in individuals without dementia. Neurology. 2008;71(12):882-7.

9. Stern Y. What is cognitive reserve? Theory and research application of the reserve concept. Journal of the International Neuropsychological Society. 2002;8(03):448-60.

10. Johansson B, Hofer SM, Allaire JC, Maldonado-Molina MM, Piccinin AM, Berg S, et al. Change in cognitive capabilities in the oldest old: the effects of proximity to death in genetically related individuals over a 6-year period. Psychology and Aging; Psychology and Aging. 2004;19(1):145.

11. Batterham PJ, Mackinnon AJ, Christensen H. The effect of education on the onset and rate of terminal decline. Psychology and aging. 2011;26(2):339.

12. Muniz-Terrera G, van den Hout A, Piccinin AM, Matthews FE, Hofer SM. Investigating Terminal Decline: Results From a UK Population-Based Study of Aging. 2012.

13. Muniz‐Terrera G, Matthews FE, Stephan B, Brayne C. Are terminal decline and its potential indicators detectable in population studies of the oldest old? International journal of geriatric psychiatry. 2011;26(6):584-92.

14. MunizTerrera G, Van den Hout A, Matthews FE. Random change point models: investigating cognitive decline in the presence of missing data. Journal of Applied Statistics. 2011;38(4):705-16.

15. Fleming J, Zhao E, O'Connor DW, Pollitt PA, Brayne C. Cohort profile: the Cambridge City over-75s Cohort (CC75C). International journal of epidemiology. 2007;36(1):40-6.

16. Folstein MF, Robins LN, Helzer JE. The mini-mental state examination. Archives of General Psychiatry. 1983;40(7):812.

17. Kiuchi AS, Hartigan JA, Holford TR, Rubinstein P, Stevens CE. Change points in the series of T4 counts prior to AIDS. Biometrics. 1995:236-48.

18. Lunn DJ, Thomas A, Best N, Spiegelhalter D. WinBUGS-a Bayesian modelling framework: concepts, structure, and extensibility. Statistics and computing. 2000;10(4):325-37.

19. Spiegelhalter DJ, Best NG, Carlin BP, Van Der Linde A. Bayesian measures of model complexity and fit. Journal of the Royal Statistical Society: Series B (Statistical Methodology). 2002;64(4):583-639.

20. Brayne C, Ince PG, Keage HAD, McKeith IG, Matthews FE, Polvikoski T, et al. Education, the brain and dementia: neuroprotection or compensation? EClipSE Collaborative Members. Brain. 2010;133(8):2210-6.

21. Matthews FE, Jagger C, Miller LL, Brayne C. Education differences in life expectancy with cognitive impairment. The Journals of Gerontology Series A: Biological Sciences and Medical Sciences. 2009;64(1):125.

22. Fries JF. Aging, natural death, and the compression of morbidity. BULLETIN-WORLD HEALTH ORGANIZATION. 2002;80(3):245-50.

23. Wilson RS, Beck TL, Bienias JL, Bennett DA. Terminal cognitive decline: accelerated loss of cognition in the last years of life. Psychosomatic Medicine. 2007;69(2):131-7.

24. Laird NM, Ware JH. Random-effects models for longitudinal data. Biometrics. 1982:963-74.

25. Thorvaldsson V, Hofer SM, Johansson B. Aging and late-life terminal decline in perceptual speed. European Psychologist. 2006;11(3):196-203.

26. Manly JJ. Deconstructing race and ethnicity: implications for measurement of health outcomes. Medical care. 2006;44(11):S10-S6.
